# Trajectory Planning for Unmanned Vehicles on Airport Apron Under Aircraft–Vehicle–Airfield Collaboration

**DOI:** 10.3390/s25010071

**Published:** 2024-12-26

**Authors:** Dezhou Yuan, Yingxue Zhong, Xinping Zhu, Ying Chen, Yue Jin, Xinze Du, Ke Tang, Tianyu Huang

**Affiliations:** 1 School of Air Traffic Management, Civil Aviation Flight University of China, Guanghan 618307, China; ftu9275@163.com (D.Y.); zzyx0109@163.com (Y.Z.); zxp@cafuc.edu.cn (X.Z.); tangke@cafuc.edu.cn (K.T.); 2 Capital Airports Holdings Co., Ltd., Beijing 101317, China; cheny3@cahs.com.cn (Y.C.); jinyue3@cahs.com.cn (Y.J.); duxinze@cahs.com.cn (X.D.); 3 Key Laboratory of Green Airport, CAAC, Beijing 101317, China

**Keywords:** air transportation, vehicle trajectory planning, multi-agent reinforcement learning

## Abstract

To address the issue of safe, orderly, and efficient operation for unmanned vehicles within the apron area in the future, a hardware framework of aircraft–vehicle–airfield collaboration and a trajectory planning method for unmanned vehicles on the apron were proposed. As for the vehicle–airfield perspective, a collaboration mechanism between flight support tasks and unmanned vehicle departure movement was constructed. As for the latter, a control mechanism was established for the right-of-way control of the apron. With the goal of reducing waiting time downstream of the pre-selected path, a multi-agent reinforcement learning model with a collaborative graph was created to accomplish path selection among various origin–destination pairs. Then, we took Apron NO.2 in Ezhou Huahu Airport as an example for simulation verification. The results show that, compared with traditional methods, the proposed method improves the average vehicle speed and reduces average vehicle queue time by 11.60% and 32.34%, respectively. The right-of-way signal-switching actions are associated with the path selection behavior of the corresponding agent, fitting the created aircraft–vehicle collaboration. After 10 episodes of training, the Q-values can steadily converge, with the deviation rate decreasing from 40% to below 0.22%, making the balance between sociality and competitiveness. A single trajectory can be planned in just 0.78 s, and for each second of training, 7.54 s of future movement of vehicles can be planned in the simulation world. Future research could focus on online rolling trajectory planning for UGSVs in the apron area, and realistic verification under multi-sensor networks can further advance the application of unmanned vehicles in apron operations.

## 1. Introduction

In recent years, techniques such as artificial intelligence, vehicle–road collaboration, and unmanned driving have matured daily. The Civil Aviation Administration of China (CAAC) is also actively promoting the application of unmanned driving technology at airports [1]. Specifically, most unmanned ground support vehicles (UGSVs) operate in the apron area, which is a designated zone for activities, such as passenger boarding and disembarking, loading and unloading mail or cargo, fueling, parking, and maintenance of aircraft. With the tendency of unmanned flight support mode, the mixed operation of UGSVs and human-driven aircraft on the apron poses challenges to operational safety and efficiency. In this context, the movement of aircraft is dense, and the occupancy time of apron resources is relatively fixed; meanwhile, the total task volume of UGSVs is large, and the operation time is incredibly flexible, but a tight relationship exists among various support tasks. Vehicle–infrastructure collaboration is a good way to manage these moving targets and enhance the level of service and safety on the apron. Therefore, what we are concerned about is how to reasonably plan the trajectories of numerous UGSVs within the framework of “aircraft–vehicle–airfield” collaboration.

Related work includes two categories: operations research optimization and simulation optimization. Optimizations based on operations research follow two approaches: scheduling for a single type of UGSVs and multiple types of UGSVs. The former focuses on the precise collaboration between a single type of vehicle and multiple parking positions, as well as the alignment of vehicle operations with flight support demands. Some studies explore the collaborative mechanisms and patterns, such as summarizing available optimization models and intelligent algorithms for the vehicle and aircraft time slot allocation problem [2]. By using grey model methods, time series methods, and machine learning techniques, researchers can accurately predict the demand for UGSVs at airports and the distribution patterns of airport flights [3]. The latter typically focuses on a single flight and addresses the scheduling problem of multiple types of support vehicles. Relevant studies include using multi-objective genetic algorithms to solve the problem between fuel trucks and shuttle buses [4]. The third generation of non-dominated sorting genetic algorithms (NSGA-III) can solve the collaborative scheduling problem of three types of UGSVs [5]. Some scholars categorize 14 types of UGSVs, summarize three operational patterns, and decompose the problem into three sub-problems about the Markov chain, employing an improved dual-layer dynamic programming (DLP) algorithm [6]. These studies demonstrate certain improvements in reducing computation time and enhancing solution accuracy.

Optimization based on simulation focuses on safety issues during the trajectory planning process. It involves discrete system modeling and simulation; through effective operational regulations and simulation validations, conflicts between trajectories can be avoided. For instance, utilizing complex network models such as GeoSOT and Petri nets to represent airport/apron movement situations, identifying and managing potential conflicts [7]. Based on the precise perception of the situation between UGSVs and aircraft, kinematic models are employed for conflict early warning in the apron area [8,9]. Regarding the conflict hotspot identification problem at airports, an improved GeoSOT model and a grey analysis system are used to categorize and manage movement conflicts among aircraft, among vehicles, and between aircraft and vehicles [10]. An elliptical protection zone is established by integrating the dimensions and motion attributes of entities for conflict prediction [11]. Furthermore, combined with an advanced surface movement guidance and control system (A-SMGCS), a physical model based on Petri nets and a conflict monitoring mechanism are created through the concepts of right-of-way control unit division and decentralized collaboration [12].

In the field of road transportation, reinforcement learning methods for intersection traffic optimization and control can provide valuable insights for this paper. For a single road intersection, a mixed model with transfer learning and deep reinforcement learning can explore the impact of different urban road network configurations on trajectory planning [13]. Combined with the vehicle platooning method and a multi-objective linear optimization model, trajectory planning for unmanned vehicles on a single road section can be implemented [14]. Deep reinforcement learning models are also used to control traffic lights at an isolated intersection [15,16]. Based on the H-PPO algorithm and traffic flow prediction, phase adaptive signal control for a single intersection can be conducted [17]. For multi-intersection traffic control problems, multi-agent reinforcement learning (MARL) can be constructed to realize collective intelligence [18]. Some scholars have proposed an improved computational efficiency using a cooperative graph-based multi-agent reinforcement learning model (CG-MARL) [19]. In the vehicle-to-everything (V2X) environment, combining fog computing frontier technology and reinforcement learning algorithms can form a control mechanism for traffic signals [20].

In the field of general aviation, how to tackle sensing and communication problems during trajectory tracking, the control and planning process of unmanned aerial vehicles (UAVs) is a hot topic. Similar to road transportation, a distributed tracking algorithm for UAV swarm has been proposed. With the help of IoT, UWB, cyber-twin system, and end-to-end communication, response time in milliseconds can be achieved, reaching a tracking ratio over 95% [21]. As for the control problem, there is also a method that combines connected and automated vehicles (CAVs) with UAVs, expanding the meaning of V2X. In this way, UAVs are used for helping ground vehicle platoon management, becoming messengers between ground vehicles [22]. When it comes to large-scale trajectory planning, an advanced communication method for UAV swarm has not been established yet. Existing methods usually apply an integrated control framework, then utilize a mini UAV swarm like DJI Tello for verification [23,24].

The above research provides references for tackling the problem we encounter, but there are two main shortcomings. Firstly, most of the articles about optimization based on operation research only have pure mathematical models, which inadequately consider the regulatory constraints during apron operations. Secondly, optimizations based on simulation have concentrated on improving theoretical models about conflict identification and classification, with few proposing conflict avoidance mechanisms. Finally, due to complexity, UAV-related work gives reasonable methods for trajectory tracking, planning, or control, which can give us incredible inspiration. However, advanced communication, like 5G, has not been used due to air limitations.

Our main contributions include:

1. Presenting a two-stage trajectory planning method in the apron area. Our approach integrates vehicle assignment and path planning, which is able to manage all kinds of UGSVs reasonably.
2. In the vehicle assignment stage, considering conflict avoidance between UGSVs and aircraft, and establishing a road right-of-way control mechanism for UGSVs around the apron, thereby achieving collaboration among aircraft, vehicles, and airfield.
3. In the path planning stage, based on the CG-MARL model, integrating human-like driving concepts and operational rules of the apron area to make UGSVs select the optimal path. CG-MARL gives a distributed planning method, which could be expanded for other operation works such as cargo workshops, unmanned mines, and even unmanned aerial vehicles (UAVs).
4. Using a novel numerical simulation–microscopic traffic flow joint simulation platform to conduct a case study. The platform can also be expanded to the whole airfield. The conventional urban traffic environment can also be added to test the connectivity among passenger, cargo, and flight.

The structure of this paper is as follows: Section 2 describes the traffic scenario and the problems we face; Section 3 offers materials and methods; Section 4 shows our results and related discussion; Section 5 concludes the research and proposes future research directions.

## 2. Problem Description

**Figure 1:**
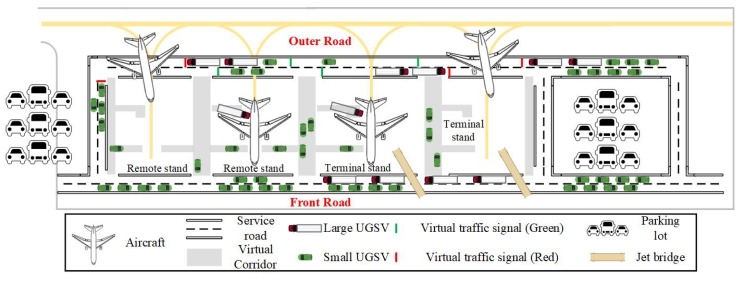
Scheme of traffic scenario in the apron area. UGSV: Unmanned Ground Support Vehicle.

The traffic scenario for UGSVs in the apron area is illustrated in Figure 1, which includes elements such as service roads, virtual corridors [25], parking stands, apron taxiways, and parking lots for UGSVs. These elements, along with UGSVs and aircraft, constitute the apron traffic system. UGSVs operate in the front or outer service road; the virtual corridors offer access for UGSVs to enter or exit parking stands. Aircraft can access the parking stands via the apron taxiways. Specifically, compared to terminal parking stands, remote parking stands require additional support from passenger boarding buses, shuttle buses, air supply vehicles, and other services.

Under this traffic scenario, we should tackle three problems:

1. How to plan the vehicle trajectory between parking lots and parking gates?
2. How to organize vehicle, aircraft, and roadside information together, and fully utilize right-of-way on the apron?
3. What kind of method is suitable for connecting the flight support tasks and vehicle movement smoothly, and avoiding potential conflicts?

We assume that:

1. The 4D taxiing trajectory of the aircraft and the parking-stand allocation scheme are known.
2. Each apron traffic system includes one vehicle parking lot at least.
3. The unmanned vehicles travel back and forth between the vehicle parking area and the parking positions.
4. Minimum spacing between the boundary of two adjacent parking stands is 4 m, allowing bidirectional traffic in the virtual corridor [25].

## 3. Materials and Methods

### 3.1. Basic Materials for Trajectory Planning

**Figure 2:**
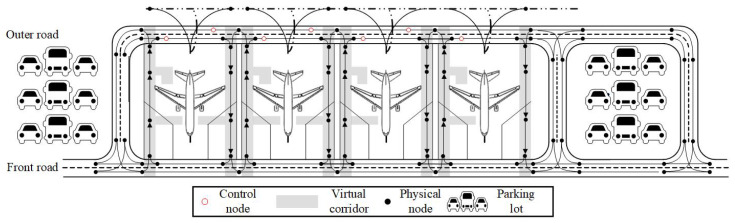
Topological diagram of apron traffic system.

Based on the node–segment topology, the model of the apron traffic system can be scientifically described as a directed connected graph G(V,E). As shown in Figure 2, the model is made up of service roads, virtual corridors, control nodes, and physical nodes. The physical nodes form the spatial contour of the apron traffic system, while the control nodes are used to manage the UGSV traffic flow on the outer road. This paper studies how to conduct trajectory planning for unmanned vehicles in this scenario. Due to the guidance from roadside equipment, UGSVs depart on time with designated O–D pairs and pass through the corresponding control nodes in order. Eventually, we can meet the collaborative needs of unmanned vehicle operations and flight support while avoiding any impact on the taxiing process of aircraft.

In response to this kind of traffic scenario, we propose an architecture of trajectory planning for UGSVs under the aircraft–vehicle–airfield collaboration.

**Figure 3:**
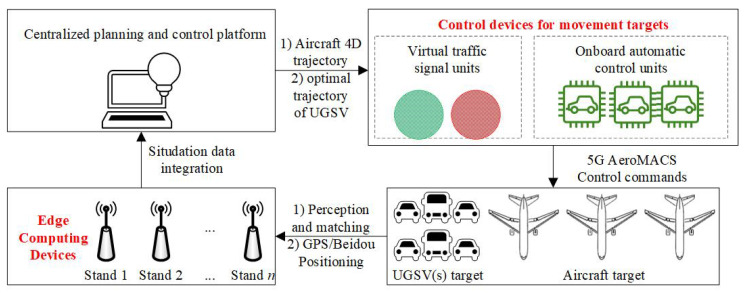
Architecture of aircraft–vehicle–airfield coordination.

As shown in Figure 3, this architecture can be divided into the following three parts:

1. **Edge computing devices in airfield,** which are responsible for edge calculations on dynamic scenes captured by roadside sensors (such as LiDAR, millimeter-wave radar, etc.). Then, situation awareness of movement targets (aircraft and UGSVs) within their designated areas can be achieved. Following this, the situational data from each edge computing device are uploaded to a centralized planning and control platform for integration, ultimately forming a comprehensive operational status of the apron traffic system.
2. **Control devices for movement targets.** This includes onboard automatic control units and virtual traffic signal units. The onboard automatic control units are distributed across the UGSVs, responsible for receiving trajectory planning results. Then, automatic control units guide the UGSVs to travel along the planned paths at a proper time window. On the other hand, the virtual traffic signal units are located at each parking position, responsible for receiving aircraft movement information. They convert right-of-way signals during the corresponding time windows of aircraft activities, making UGSVs give way to aircraft.
3. **The 5G AeroMACS network,** a next-generation aviation broadband communication technology, applies fifth-generation mobile communication technology (5G) to the dedicated civil aviation network (AeroMACS). The 5G AeroMACS (Aviation 5G System) has significant advantages in civil airport and aviation operations. Firstly, 5G AeroMACS provides low latency and high reliability communication, ensuring real-time and accurate information sharing among the cockpit, tower, ground vehicles, airlines, and airports, improving coordination and efficiency in airport operations. Secondly, the high bandwidth of 5G supports large-scale data transmission, enabling the transfer of high-precision digital maps, navigation information, real-time positions of aircraft and ground vehicles and runway and taxiway conditions, thus enhancing information sharing and decision-making efficiency.

Specifically, 5G AeroMACS has significant advantages in civil airport and aviation operations. Firstly, it provides low latency and high reliability communication, ensuring real-time and accurate information sharing among the cockpit, tower, ground vehicles, airlines, and airports. Secondly, the high bandwidth of 5G supports large-scale data transmission, enabling the transfer of high-precision digital maps, navigation information, real-time positions of aircraft and ground vehicles, and runway and taxiway conditions, thus enhancing information sharing and decision-making efficiency. Thirdly, it uses dedicated aviation frequencies, completely isolated from public networks, and meets the safety communication standards of the International Civil Aviation Organization (ICAO).

### 3.2. Control Logic of Trajectory Planning for UGSVs in the Apron Area

**Figure 4:**
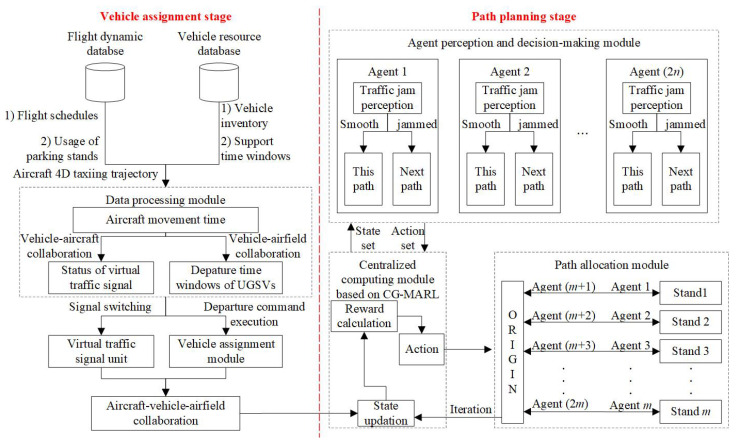
Framework of trajectory planning on the apron. CG-MARL: Collaborative Graph—Multi-Agent Reinforcement Learning.

Afterwards, we propose a two-stage trajectory planning scheme. The first stage, vehicle assignment, aims to clarify the time windows for various types of UGSVs, ensuring a smooth connection with flight support requirements. The second stage, path planning, makes the control system select suitable routes for the UGSVs based on real-time traffic conditions. The details of the two stages are shown in Figure 4, and concrete control logic is shown as follows:

1. **Vehicle Assignment Stage.** Based on the type of UGSVs and taking flight support nodes as a reference, the departure time windows of UGSVs are determined. Eventually, each unmanned vehicle executes the departure instruction. Firstly, the flight dynamic database stores the flight schedules, usage situation of parking stands, and 4D taxiing trajectories of aircraft in the future. Meanwhile, the vehicle resource database updates and stores the inventory of each category of UGSVs and the time windows of flight support in real time. All information is sent to the data processing module. This module ensures that there is no potential conflict between UGSVs and aircraft while orderly linking the departure time windows of unmanned vehicles with flight support operational nodes. Within the time windows of aircraft taxiing and UGSV operations, the virtual traffic signal unit and the vehicle assignment module will execute signal switching and departure instructions, respectively. Finally, vehicle assignment is completed, forming a basis for aircraft–vehicle–airfield collaboration.
2. **Path Planning Stage.** This includes three modules: centralized computing, agent perception and decision-making, and path allocation. In the centralized computing module, the central computer collects current performance indicators of the apron traffic system as a state set. Subsequently, based on the CG-MARL model, it calculates the reward values for different path action sets and selects the best path decision. The agent perception and decision-making module obtains the traffic state from the centralized computation module, configures agents for each O–D pair, and outputs an action set. The action set is then transmitted to the path allocation module, which allocates specific paths for the groups of UGSVs on each O–D pair. In the flight dynamic database, agents will learn and iterate over multiple rounds. Throughout this process, agents will try their best to avoid bottlenecks downstream of the path and tend to select routes that allow for smooth driving.

The technical details of the two-stage planning process, such as the collaboration mechanisms, CG-MARL model design, and database samples, are as follows.

### 3.3. Vehicle Assignment Process Under Aircraft–Vehicle–Airfield Collaboration

Vehicle assignment is based on the time windows for aircraft behaviors, such as entering or leaving their parking stands, forming an environment of aircraft–vehicle–airfield collaboration in the apron area. Let the flight dynamic database be denoted as the set $F=\{\left(I,f_{n},t_{n},\phi \left(f_{n}\right)\right)\}$, where $I={\{}^{‘}{\mathrm{Arr}}^{’}\;\;\;\;\;{,}^{‘}{\mathrm{Dep}}^{’}\;\;\;\}$ represents the arrival/departure status of the specific flight; $f_{n}$ is the identifier for the *n*-th flight; $t_{n}$ is the moment when ground crew places/displaces the wheel chock for the *n*-th flight; and $\phi (f_{n})$ is the parking stand occupied by the *n*-th flight.

Let the vehicle resource database be denoted as the set $V=\{\left(C_{i,j},T_{w1},T_{w2}\right)\}$, where $C_{i,j}$ indicates the scale of the *j*-th category of UGSVs required to support at the parking stand type *i*; $T_{w1}$ and $T_{w2}$ represent the time windows for UGSVs to enter and leave the parking stand, expressed as interval $\left[t_{\min},t_{\max}\right]$. $t_{\min}$ and $t_{\max}$ denote the earliest and latest moments relative to a reference time $t_{n}$. These two values relate to the various flight support time nodes for each type of UGSV.

#### 3.3.1. Relationship of Vehicle–Airfield Collaboration

The departure time windows for UGSVs are communicated through roadside equipment shown in Figure 3 and sent to various UGSVs, thus achieving "vehicle–airfield" collaboration. The vehicles have two optional routes: one is to depart from the parking lot to the corresponding parking stand, and the other is to go back to the parking lot from the parking stand. The departure time windows for these two routes are denoted as $T_{d1}$ and $T_{d2}$, respectively. The relationships between this kind of time window and $T_{w1}$, $T_{w2}$ are defined by the following equations:

(1) $$\left\{\begin{array}{c} T_{d1}=T_{w1}-\left(t_{\phi (f_{n})}+\delta \right)\\ T_{d2}=T_{w2} \end{array}\right..$$

Among them, $t_{\phi (f_{n})}$ represents the operation time for UGSVs from the parking lot to the parking stand $\phi (f_{n})$ (not considering the waiting time of UGSVs); δ is the backup time for waiting, mainly considering the time delay of UGSV waiting for the aircraft on the outer service road, with specific waiting methods described in Section 3.3.2. At the same time, $T_{w1},T_{w2}$ vary with the type of parking stand (*i*) and type of vehicle (*j*), distinguished as ${\left(T_{w1}\right)}_{i,j},{\left(T_{w2}\right)}_{i,j}$.

In conclusion, for the movement of UGSVs with $C_{i,j}$ passenger car units (PCUs), there is a mapping relationship of collaboration between the flight schedule and departure time window:

(2) $$F\overset{f_{1}}{\to }\left({\left(T_{d1}\right)}_{i,j},{\left(T_{d2}\right)}_{i,j}\right)$$

**Figure 5:**
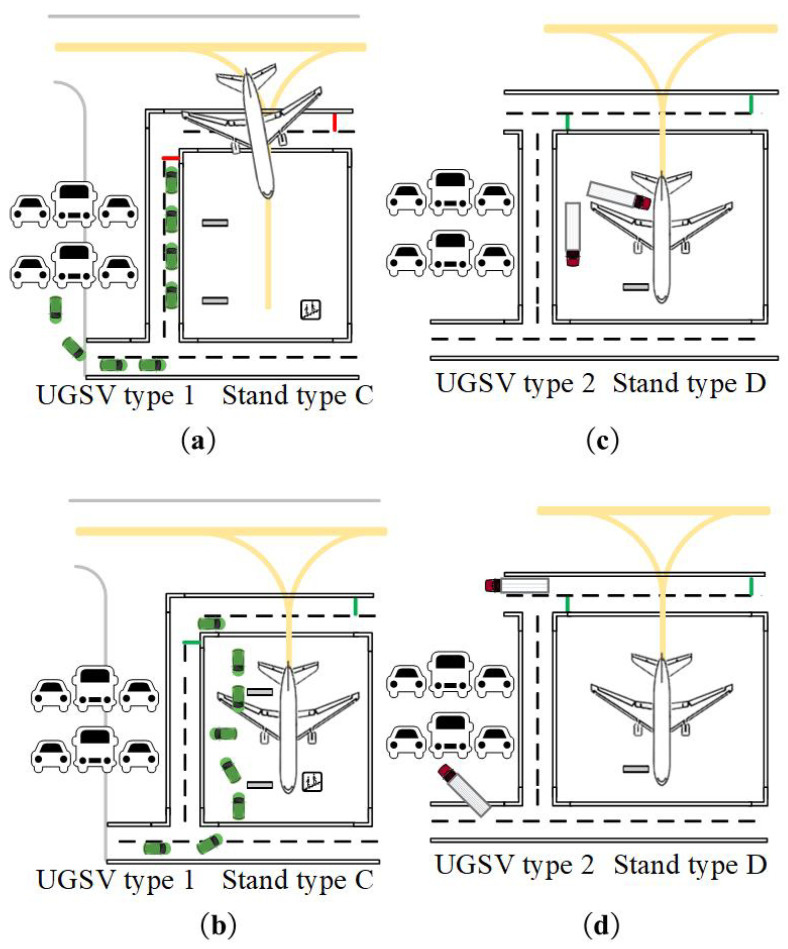
Coordination rule between aircraft and unmanned vehicles. (**a**) $t\in {\left(T_{d}^{\mathrm{in}}\right)}_{1}^{C}$, departing. (**b**) $t\in {\left(T_{w}^{\mathrm{in}}\right)}_{1}^{C}$, get to the parking stand. (**c**) $t\in {\left(T_{d}^{\mathrm{out}}\right)}_{2}^{D}$, departing. (**d**) $t\in {\left(T_{d}^{\mathrm{out}}\right)}_{2}^{D}+t_{\phi \left(f_{n}\right)}+\delta$, get to the parking stand.

Through this relationship of collaboration, the vehicle assignment module can obtain the departure time windows for all types of UGSVs. This allows it to execute dispatch commands within the given time windows, making each vehicle set out at a proper time. The effect of the scheduled assignment for UGSVs is shown in Figure 5.

#### 3.3.2. Slot Allocation for Departure of Unmanned Vehicles

For arrival flights, based on the 4D taxiing trajectory of aircraft, it is known that the aircraft takes $t_{1}$ time to move from the physical node on the taxiway into the parking stand. Let the set $R_{1}=\left\{\left(\phi \left(f_{n}\right),\left[t_{n}-t_{1},t_{n}\right]\right)\right\}$ denote the time window for the status switch of the virtual traffic signal near the parking stand $\phi (f_{n})$. The corresponding signal switches to “red light” status at time $\left(t_{n}-t_{1}\right)$, closing the right-of-way for the outer service road, so that UGSVs must wait outside the stop line. As long as it is time $t_{n}$ to place wheel chock for aircraft, the signal switches to “green light” status, releasing the right-of-way, allowing UGSVs to passage again.

For departure flights, it is known that the aircraft takes time $t_{2}$ to push back or taxi out from the parking stand to the physical node of the taxiway. Let the set of time windows for the virtual traffic signal to switch status be defined as $R_{2}=\left\{\left(\phi \left(f_{n}\right),\left[t_{n},t_{n}+t_{2}\right]\right)\right\}$. In this set, the signal switches to “red light” status at time $t_{n}$ for displacing the wheel chock; it switches to “green light” status at $\left(t_{n}+t_{2}\right)$.

In conclusion, there is a mapping relationship of collaboration between the flight schedule and the switch of the virtual traffic signal:

(3) $$F\;\;\overset{f_{2}}{\to }\;\;\left\{\begin{array}{c} R_{1},\;\mathrm{if}\;I=\;‘\mathrm{Arr}’\\ R_{2},\;\mathrm{if}\;I=\;‘\mathrm{Dep}’ \end{array}\right.\;.$$

**Figure 6:**
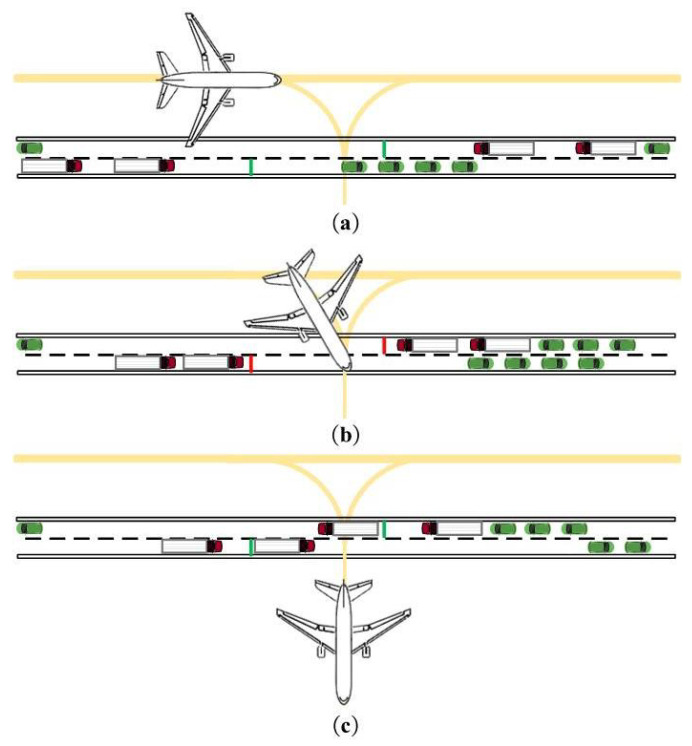
Relationship of vehicle–aircraft collaboration. (**a**) $t<t_{n}-t_{\mathrm{taxi}}^{\mathrm{in}}$. (**b**) $t_{n}-t_{\mathrm{taxi}}^{\mathrm{in}}⩽t⩽t_{n}$. (**c**) $t>t_{n}$.

Through this relationship of collaboration, the virtual traffic signals can accurately identify whether the aircraft are occupying the outer service road in the apron area. This allows for the timely closure and release of right-of-way, achieving vehicle–aircraft collaboration. If we take the current time as *t* and use the example of an arriving aircraft taxiing into a parking stand, Figure 6 presents the expected effects of vehicle–aircraft collaboration.

### 3.4. Path Planning for UGSVs in the Apron Area Based on CG-MARL Model

This section provides a path planning method for UGSVs based on the CG-MARL model under the aircraft–vehicle–airfield collaboration. Through multi-agent learning and training, UGSVs can exhibit human-like driving behaviors, ultimately achieving the path planning function.

In previous studies, Q-learning algorithms are commonly used methods for solving traffic problems with MARL models due to their historical experience memory function, simplicity, and ease of deployment [26]. The essential idea is to perceive the uncertain elements in the environment and learn the optimal strategy. Subsequently, actions are executed in a specific environment based on this strategy, leading to state transitions and the acquisition of reward signals from the environment. Ultimately, the relationship between states, optimal actions, and reward signals is stored in a Q-table of size $\left(N_{s}\times N_{a}\right)$. Where $N_{s}$ and $N_{a}$ represent the number of elements in the state space and action space, respectively. The agent’s cognition and learning about the environment are continuously reinforced through Q-table iterations until convergence, indirectly yielding the optimal strategy [20]. The elements in the Q-table are referred to as Q-values. The Q-value update method is as follows:

(4) $$Q_{t+1}\left(s_{t},a_{t}\right)=Q_{t}\left(s_{t},a_{t}\right)+\alpha \left[r_{t}\left(s_{t},a_{t}\right)+\gamma Q_{t}\left(s_{t},a_{t}\right)\right].$$

where $s_{t}$ and $a_{t}$ represent the state and the chosen action at time *t*; $r_{t}\left(s_{t},a_{t}\right)$ denotes the reward obtained from the environment corresponding to the action and state at time *t*. α and γ are common parameters in learning systems, representing the learning rate and discount factor, with values in the range of [0,1].

Unlike traditional MARL models, the CG-MARL model matches the O–D pairs with each agent one-to-one. By utilizing a collaborative graph strategy, it clarifies the interaction relationships among agents and the environment, states, and actions within the learning system. Then, we can reduce variable dimensions and improve computational efficiency.

**Figure 7:**
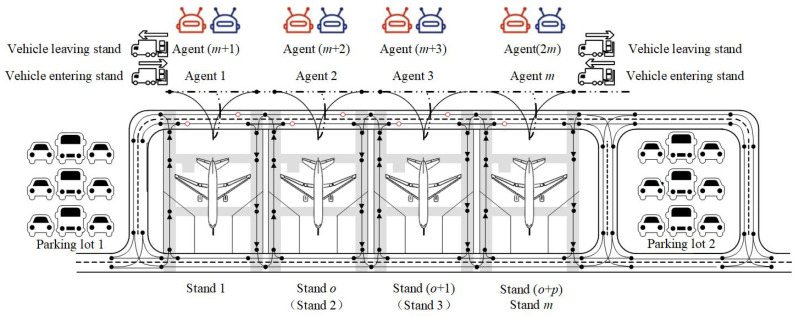
Scheme for communications on the apron (o=2, p=2 in this case).

In the CG-MARL model, the definitions are as follows:

**Agents.** As shown in Figure 7, each parking stand corresponds to two agents, managing two O–D pairs. Two O–D pairs dominate the group of UGSVs entering and exiting the parking stand, respectively. Let the number of parking positions be *m*, resulting in a total of 2m agents, denoted as Agent(1)∼Agent(2*m*). Where Agent(1)∼Agent(*m*) corresponds to *m* O–D pairs for entering the parking stands, while Agent(*m* + 1)∼Agent(2*m*) corresponds to the remaining *m* O–D pairs for exiting the parking stands.

**Figure 8:**
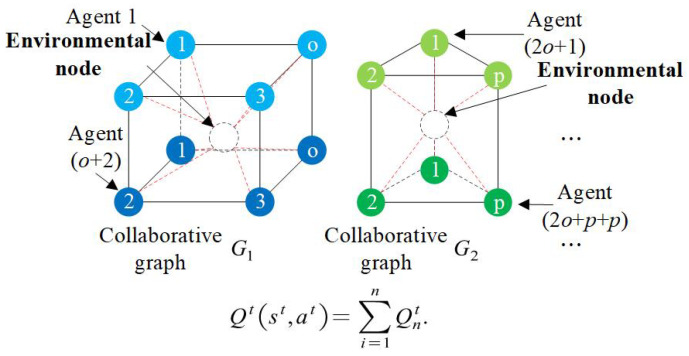
Collaborative graph for reinforcement learning.

**Collaborative graph.** Represented as a non-directional connected graph $G_{k}\left(V_{A},E_{I}\right)$, indicating a set of agents with interactive relationships. The nodes $V_{A}$ represent the various agents, while the edges $E_{I}$ indicate the interaction of states and actions among the agents. As shown in Figure 7 and Figure 8, the parking lot *k* and related agents correspond to the collaborative graph $G_{k}$. All agents receive reward signals from the central environmental node. The upper and lower layer nodes of the collaborative graph represent the agents entering and leaving the parking stands, respectively. Taking the collaborative graph $G_{1}$ as an example, the upper layer nodes correspond to the parking stands (1∼o) near parking lot 1. Meanwhile, Agent(1)∼Agent(*o*) matching *o* O–D pairs that enter the parking stand. For the lower layer nodes, they correspond to Agent(*o* + 1)∼Agent(2*o*), which match *o* O–D pairs that leave the parking stand.

**Action space.** We mainly consider imitating the path selection behavior of human drivers, leading agents to avoid congestion and choose the optimal route. In this traffic scenario, there are two types of path selection behaviors, that is

(5) $$a=\left\{\begin{array}{c} P_{1},\;agent\;choose\;the\;front\;road;\\ P_{2},\;agent\;choose\;the\;outer\;road. \end{array}\right.$$

Each intelligent agent is responsible for multiple autonomous vehicles on a single O–D pair at the current moment and conducts path selection. As shown in Equation (5), each agent has two action choices. For example, for Agent(1), the action space set is given by $A_{1}=\left\{P_{1},P_{2}\right\}$.

**State space.** Considering the needs of human-like driving, it consists of the combination of current time and the congestion conditions of each path’s downstream intersections. Each agent observes whether congestion occurs downstream on their respective paths $P_{1}$ and $P_{2}$. There are four combinations of congestion conditions:

(A) (0,0), indicating that neither of the two available paths has congestion downstream;
(B) (0,1), indicating that path $P_{1}$ has no congestion downstream, but path $P_{2}$ has congestion downstream;
(C) (1,0), indicating that path $P_{1}$ has congestion downstream, but path $P_{2}$ has no congestion downstream;
(D) (1,1), indicating that both available paths have congestion downstream.

Queue length is a commonly used congestion determination indicator in the field of road traffic, and it can be easily read through autonomous driving simulators [27]. Furthermore, considering the relativity of congestion, we use the average queue length rather than a constant as the threshold to determine congestion conditions. First, let the set of queue lengths at all intersections downstream of path *k* at the current time *t* be denoted as $L_{t,k}$, and take the maximum element from the set. Next, compare $L_{t,k}$ with the average queue length $\bar{l_{t}}$ of the apron traffic system at the current time *t*. Finally, if $L_{t,k}>\bar{l_{t}}$, then it is determined that congestion occurs on the path. The judging condition is shown as Equation (6):

(6) $$\max\left(L_{t,k}\right)>\bar{l_{t}},\;k\in P_{l}\;\left(l=1,2\right).$$

Therefore, ∀Agent(M)(M=1,2,⋯,2m), the state space can be set as in the following equations:

(7) $$S_{M}=\left\{\left(x,y\right)|\left(0,0\right),\left(0,1\right),\left(1,0\right),\left(1,1\right)\right\}.$$

(8) $$x=\left\{\begin{array}{c} 0,\;\mathrm{if}\max\left(L_{t,P_{1}}\right)\leq \bar{l_{t}}\\ 1,\;\mathrm{if}\max\left(L_{t,P_{1}}\right)>\bar{l_{t}} \end{array}\right.\;;$$

(9) $$y=\left\{\begin{array}{c} 0,\;\mathrm{if}\max\left(L_{t,P_{2}}\right)\leq \bar{l_{t}}\\ 1,\;\mathrm{if}\max\left(L_{t,P_{2}}\right)>\bar{l_{t}} \end{array}\right.\;.$$

**Reward function.** The reward function is set as the reciprocal of the average queue length of the apron traffic system at the current moment. Considering that the lower bound for the queue length is 0, a translation adjustment is applied to the independent variable of the reward function. Furthermore, we also normalize the indicator as follows:

(10) $$R_{t}=\frac{1}{\bar{l_{t}}+1}.$$

where describes the calculation method for the instantaneous reward $R_{t}$ at the current time *t*.

Therefore, in a single round of simulation, we can calculate the cumulative reward as follows:

(11) $$R_{\mathrm{sum}}=\sum_{t^{*}=1}^{t}R_{t^{*}}=\sum_{t^{*}=1}^{t}\frac{1}{\bar{L_{q,t^{*}}}+1}.$$

### 3.5. Simulation Platform

**Figure 9:**
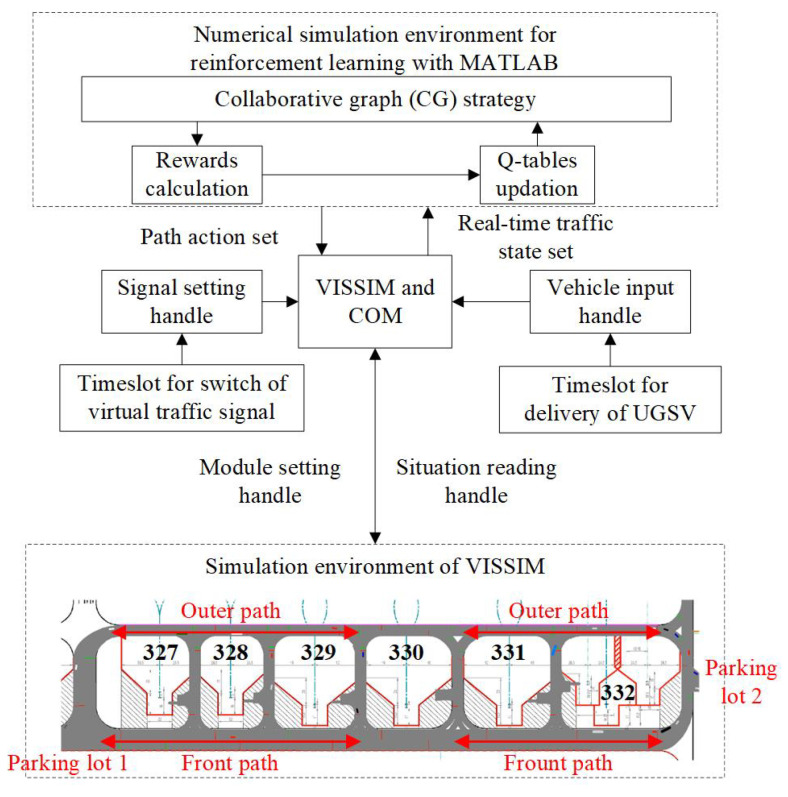
Data flows of MATLAB–VISSIM co-simulation platform.

This section utilizes the microscopic traffic simulation software VISSIM and its COM interface to establish a MATLAB–VISSIM joint simulation platform. The data flow of the platform is shown in Figure 9.

The VISSIM 4.30 software and its COM interface serve as a link between the MATLAB R2022b numerical simulation environment and the VISSIM traffic simulation environment. Specially, VISSIM 4.30 is an old version making our method universal, while MATLAB is R2022b to support reinforcement learning and comparisons with neutral networks. By using handles to set signal timing and vehicle input, VISSIM provides a foundation for simulation under aircraft–vehicle collaboration. Through module setting and situation reading handles, data flow between MATLAB and VISSIM is built. Numerical simulations and traffic simulations are conducted synchronously, allowing agents to read real-time state information within each collaborative graph. Furthermore, by creating a unified traffic environment, reward signals can be obtained, and path decisions can be executed, enabling agents to negotiate with one another.

## 4. Case Study

### 4.1. Experimental Settings

#### 4.1.1. Case Information

The experimental scenario is apron No. 2 at Ezhou Huahu Airport. Ezhou Huahu Airport is the first airport utilized for large-scale verification of unmanned driving in the airfield area in China. Under the airport surface, 50,000 sensors were equipped, forming the hardware basis of the aircraft–vehicle–airfield collaboration. Although it is an airport for cargo transportation, some passenger transportation has been operating, and all the flights with passengers onboard are operated on apron NO.2. Therefore, this apron includes two categories of flights. Additionally, this apron has a linear physical structure, which is the most conventional apron structure in the field of civil aviation, so it is representative.

As shown in the “Simulation environment of VISSIM” section of Figure 9, there are six parking stands in the apron area, numbered 327 to 332 from left to right. Among these, stand 332 is classified as an F-type, stands 329 to 331 are E-type, and the remaining stands are D-type. At both ends of the apron, there are parking lots designated, which align with the traffic scenario discussed in this paper.

#### 4.1.2. Parameter Settings

For the numerical parameters, we refer to the “Flight Safety Operation Support Standards” of CAAC, the survey results of flight support nodes of time in the apron area, and previous values set in the literature on flight support nodes [4,6,28]. Eventually, a dataset where each stand undergoes an average of two rounds of turnaround service was generated. The dataset corresponds to a VISSIM simulation duration of 12,000 s. The simulation accuracy was set to one time step. To avoid conflicts among UGSVs, the right-of-way at intersections of service roads in the apron area were controlled, referencing the concept of “rhythmic traffic” [29,30,31]. The average time for an aircraft to taxi into the stand was set as $t_{1}$, and the time for taxiing out/pushing back from the stand was set as $t_{2}$. Each of them was set with an average value of 3 min [32].

For the signal timing and vehicle input in VISSIM, the settings refer to results obtained during the vehicle assignment phase. In this phase, the flight schedule database and vehicle resource database information were first collected to form the basic data; then, through the collaborative relationships shown in Equations (2) and (3), the basic data were converted into the corresponding time window allocation results. The basic data and time window data samples are illustrated in Table 1 and Table 2, where Table 1 shows a sample of basic vehicle data, and Table 2 displays a sample of basic flight data and time window data. In the MATLAB reinforcement learning section, the learning system is set with a learning rate of α=0.6 and a discount factor of γ=0.5. A ε-greedy strategy is employed to explore the action set, with ε=0.3 [33,34].

### 4.2. Results and Related Discussion

#### 4.2.1. Training Performance of Agents

**Figure 10:**
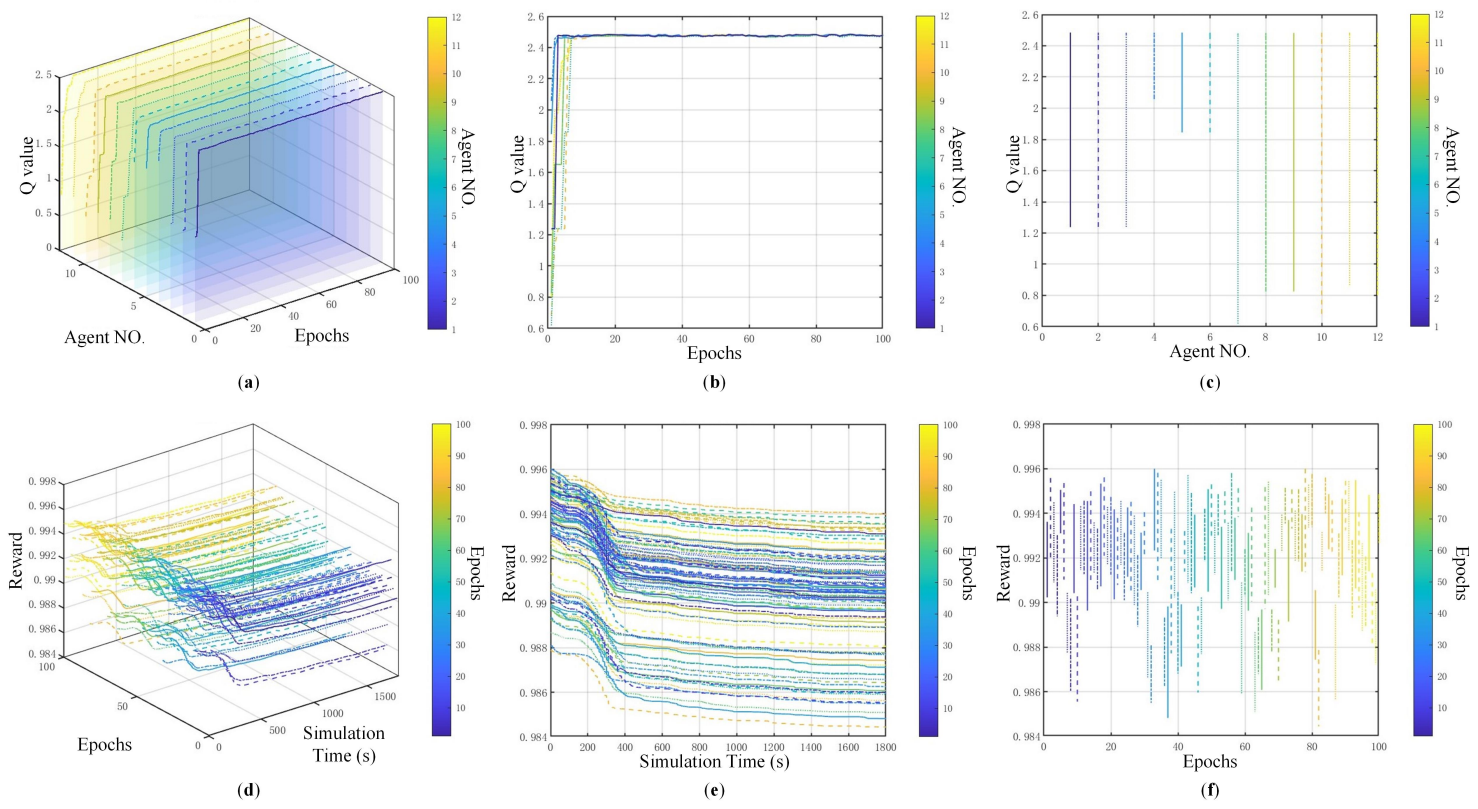
Training results of multiple agents by CG-MARL model. (**a**) The 3D graph of the convergence situation of various agents. (**b**) Q value-training epoch curve. (**c**) Distribution of Q-value of each agent. (**d**) The 3D graph about reward-related convergence situation of each simulation. (**e**) Reward–simulation time curve. (**f**) Distribution of reward of each simulation.

After 100 training episodes, all 12 agents in this example stabilized and demonstrated social behavior. As shown in Figure 10a,b, the Q-values converge to the interval [2.47, 2.50], with all agents reaching a stable state by the 10th episode. The earliest and latest convergence times for agents are 2 and 8 episodes, respectively, with the curves of all agents showing high consistency. Figure 10c indicates that the starting Q-values of agents were in the range of [0.6, 2.1], grouped from low to high as agents 7–9, 10–12, 1–3, and 4–6. Although there is a maximum initial difference of 1.5 in Q-values among agents, all converge within the target range after training. This, along with the highly consistent curve shapes, suggests that CG-MARL training enables agents to quickly develop social behavior. Compared to agents entering the parking stand executing support tasks, agents 7–12 leaving the stand receive lower rewards, corresponding to a longer average queue length and higher traffic density. Conversely, agents 4–6 and 7–9 on the right side of the apron (stand 330–332) receive higher rewards, aligning with lower average queue lengths and less traffic density. Figure 10c reflects the consistant layout and relative positioning situation between the parking lots and parking stands.

In the original 20 epochs, as the simulation progresses, the system can explore higher rewards, with the average increasing from 0.992 to 0.994 (according to Equation (10)). A reward value of 0.994 corresponds to an average queue length of 0.006 m at intersections of the service road, resulting in a cumulative queue length of 72.435 m over 12,000 s. After that, the rewards gradually decline and begin to cycle. Specifically, in the first 50 simulations represented by the blue section in Figure 10d,e, the reward values are concentrated in the range of [0.991, 0.993], overall exceeding 0.99, belonging to a moderately high level. During some training sessions, the reward values drop below 0.988, with individual instances decreasing to the range of [0.984, 0.985]. Figure 10f shows that the differences in reward values across simulations are relatively small, but as training progresses, the system tends to achieve higher rewards. Comparing all reward values, the lowest is close to 0.984, occurring at the 83rd simulation, while the highest is 0.996, occurring at the 77th simulation. Using a reward value of 0.99 as a boundary, the average reward value over 21 simulations is lower, with 11 of these occurring within the first 50 simulations. The number of simulations with reward values greater than 0.99 is similar in both the first 50 and the last 50 simulations, with 38 and 40, respectively.

**Figure 11:**
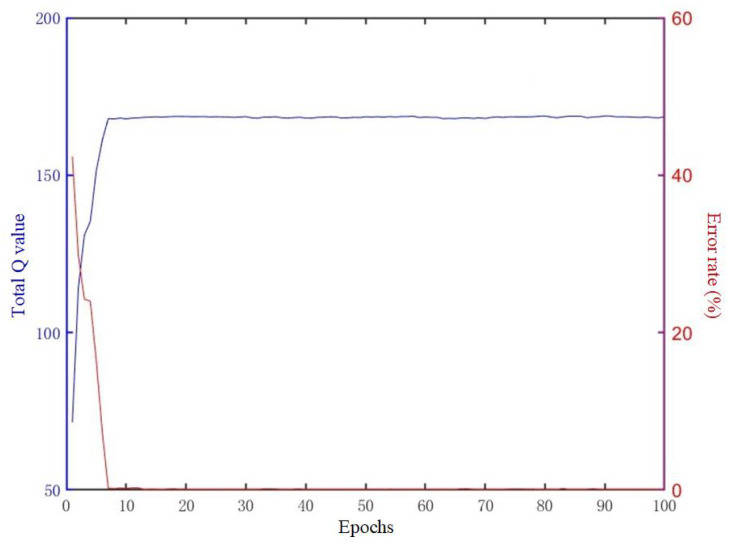
Convergence curve of total Q value of system and error rate.

The total Q-value of the system shown in Figure 11 stabilizes and converges within 10 iterations, consistent with the performance of each agent. Initially, the Q-value error rate of different agents is relatively high, exceeding 40%. That is because the ε-greedy strategy makes agents randomly select their paths in the beginning. Hence, the initial action of each agent is quite different, indicating the competitiveness among agents. The competitiveness can also be found in Figure 7 and Figure 8: agents from the same collaborative graph have public paths. As the training progresses and the Q-values stably converge, the error rate approaches 0, falling within the range of [0.0751%, 0.2184%], reflecting that the sociality of agents can be learned from the central environmental node of the collaborative graph.

#### 4.2.2. Collaboration and Planning Performance

Based on the results of the reward and Q-value of the system, setting the training rounds to 10 is appropriate in this case. Therefore, the following experiments will adjust the training rounds to 10, further comparing the performance of how agents interact and collaborate with the environment. Then, we evaluate trajectory planning performance under CG-MARL and other different algorithms.

**Figure 12:**
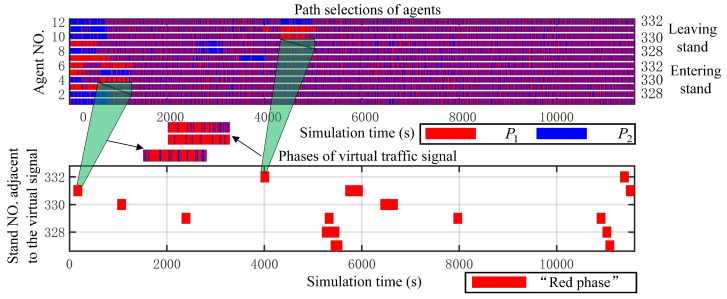
Gantt chart for path selections of agents and phases of virtual traffic signal.

As shown in Figure 12, a joint comparison of the path selection of various agents and the time-varying characteristics of virtual traffic signals indicates a close coupling between them. In this way, we succeed in making a collaborative mechanism for the path planning of UGSVs under the aircraft–vehicle–airfield collaboration. For example, at stand 331 and its corresponding agents Agent(4) and Agent(8) controlling vehicles group entering and exiting the stand, respectively, when the virtual traffic signal turns “red”, the adjacent control nodes of the corresponding stand begin to manage the right-of-way. These agents concentrate on selecting path $P_{1}$ during the intervals [200 s, 1000 s] and [4200 s, 5000 s]. The concentration of agents selecting $P_{1}$ corresponds to a relatively dense red area, indicating that similar linked features exist during other time periods. However, during the remaining time periods, the red areas do not concentrate into patches, primarily due to the absence of vehicle departures during certain periods, allowing agents to still retain the option of choosing $P_{2}$. In summary, the proposed method in this paper enables each agent to select suitable paths based on the downstream queuing situation. It makes smooth traffic flow in the apron area.

**Figure 13:**
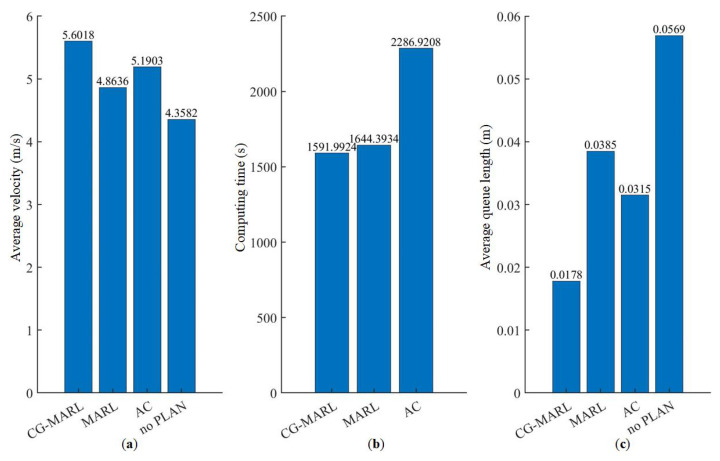
Indicator comparisons between different methods for trajectory planning. (**a**) Average velocity. (**b**) Computing time. (**c**) Average queue length.

Under conditions of no planning, the newest planning method based on actor–critic (AC) [35], traditional MARL, and the proposed CG-MARL framework, three kinds of baseline metrics, the average vehicle velocity, the computing time, and the average vehicle queue length are represented in Figure 13. Additionally, vehicle velocity and vehicle queue length are widely utilized to measure efficiency in the field of autonomous driving, which is especially suitable for the validation of VISSIM and artificial intelligence approaches [26]. In each experiment, we used the timer function from MATLAB to acquire computing time from when the training process begins to when it ends. Afterwards, we can load the velocity of each vehicle by a trajectory file exported by VISSIM. The average queue length can be calculated by the queue counters from VISSIM as well.

More specifically, in terms of average vehicle velocity, the proposed method improves by 28.53% and 11.60% compared to the no-planning and traditional MARL methods, respectively. Meanwhile, The vehicle velocity of our method is 7.93% higher than that of AC. As for computing time, the proposed method increases computational speed by 3.19% compared to traditional MARL. However, the computing time of AC is 2286.9208 s, 43.65% higher than that of CG-MARL. As for the average queue length, the CG-MARL method reduces it by 68.72% and 32.34% compared to the no-planning and traditional MARL methods, respectively. The average queue length of AC is 0.0315 m, retaining a medium level between MARL and CG-MARL. In summary, the proposed method demonstrates significant optimization effects in improving average vehicle speed, reducing queue length and minimizing computing time.

**Figure 14:**
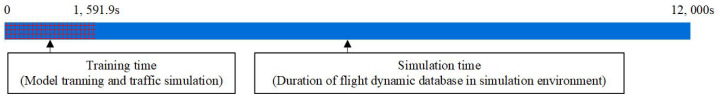
Comparison between training time and simulation time.

The results of computing time in CG-MARL show that we have the ability to plan 2035 trajectories in just 1591.9 s. In other words, a single trajectory can be planned in just 0.78 s. According to the flight dynamic database shown in Table 1 and Table 2, all the trajectories are scheduled to appear on the apron in 12,000 s. Therefore, as shown in Figure 14, the time ratio of simulation to training is 12,000:1591.9 (7.54:1). In short, for each second of training, 7.54 s of future movement of vehicles can be planned in the simulation world. Due to the ability of communication within 20 ms by 5G AeroMACS and the universality of the experimental apron, the time ratio of actual planning to training would be less than 8:1 under the same computing level.

## 5. Conclusions

1. Under the conditions of introducing 5G AeroMACS and edge computing technology, an aircraft–vehicle–airfield collaboration mechanism has been established to effectively manage the movement of UGSVs and aircraft in the apron area.
2. A trajectory planning method for UGSVs based on the CG-MARL model has been proposed. By calculating the time windows of flight activities, this method precisely aligns between UGSVs’ movement and flight support needs. Through a customized collaborative graph and a human-like driving concept, the interaction between multiple agents and the apron traffic environment is optimized. The MATLAB–VISSIM joint simulation platform has been built to provide a practical tool for the proposed method.
3. In the future, further consideration can be given to the online trajectory planning requirements of UGSVs in the apron area, proposing a rolling online planning method for scenarios with varying flight density gradients. Additionally, research on the realistic vehicle verification of unmanned driving techniques under multi-sensor networks can be conducted to further promote the application exploration of unmanned driving equipment in the apron area.

## Figures and Tables

**Table 1:** Sample of basic vehicle data. PCU: passenger car unit.

Vehicle Type	Required Traffic Volume for Single Flight (PCU)	Time Window Entering Stand	Time Window Leaving Stand
1 (Large UGSV)	5 (Stand type C) 10 (Stand type D) 10 (Stand type E) 15 (Stand type F)	**[−59, −39]**	**[−29, −9]**
2 (Small UGSV)	50 (Stand type C) 70 (Stand type D) 70 (Stand type E) 90 (Stand type F)	**[−89, −37]**	**[−52, −22]**
…	…	…	…

**Table 2:** Sample of basic flight data and time window data. DTW: departure time window.

Flight Number	Block-In Time	Block-Out Time	Stand	“Red Light” Phase Time Window	DTW 1 (Parking Lot)	DTW 1 (Stand)	DTW 2 (Parking Lot)	DTW 2 (Stand)	…
1	4:00	5:30	330	[3:57, 4:00] and [5:30, 5:33]	[4:28, 4:57]	[5:01, 5:20]	[3:58, 4:49]	[4:38, 5:07]	…
2	3:51	5:21	331	[3:48, 3:51] and [5:21, 5:24]	[4:19, 4:38]	[4:52, 5:11]	[3:49, 4:40]	[4:29, 4:58]	…
3	3:19	5:19	332	[3:16, 3:19] and [5:19, 5:22]	[4:17, 4:36]	[4:50, 5:09]	[3:47, 4:38]	[4:27, 4:56]	…
…	…	…	…	…	…	…	…	…	…

## Data Availability

The authors confirm that the data supporting the findings of this study are available within the article. The data presented in this study are available on request from the corresponding author due to privacy.

## References

[1] CAAC (2021). Roadmap for Application of Airport Unmanned Driving Equipment (2021–2025).

[2] Zhang H. (2021). Research on Airport Time Slot Allocation Technology in Ground Holding Policy. Master’s Thesis.

[3] Yang F. (2021). Demand Forecast of Ground Service Support Equipment in Hub Airport Considering Flight Delays. Master’s Thesis.

[4] Feng X., Ren Z. (2016). Collaborative scheduling of fuelling vehicle and ferry vehicle based on genetic algorithm. J. Transp. Syst. Eng. Inf. Technol..

[5] Wu Z. (2022). Research on Collaborative and Dynamic Scheduling of Ground Support Vehicles for Large Airports. Master’s Thesis.

[6] Tabares D., Mora-Camino F., Kahraman C., Aydin S. (2022). Intelligent and fuzzy applications in aircraft handling services with Aviation 4.0. Intelligent and Fuzzy Techniques in Aviation 4.0: Theory and Applications.

[7] Luo J. (2018). The Conflict Control Theory and Simulation Verification of the Apron Activity Target.

[8] Zhang T., Zhu X., Li J., Chen H., Li Z. (2023). Research on conflict detection model for taxi-in process on the apron based on aircraft wingtip keypoint detection. IET Intell. Transp. Syst..

[9] Zhang T., Zhang Z., Zhu X., Chen B., Li J., Zhong Y. (2024). Aircraft engine danger areas incursion detection using keypoint detection and IoT. Alex. Eng. J..

[10] Wang X., Zhou D. (2023). Identification of critical grid areas in flight area conflicts based on complex network. J. Saf. Sci. Technol..

[11] Yuan D., Zhu X., Zou Y., Zhao Q. (2024). Integrated optimization of scheduling for unmanned follow-me cars on airport surface. Sci. Rep..

[12] Zhu X. (2014). Research on Petri Nets-based Aircraft routing and conflict control for A-SMGCS.

[13] Lee D. (2024). Transfer Learning-Based Deep Reinforcement Learning Approach for Robust Route Guidance in Mixed Traffic Environment. IEEE Access.

[14] Feng H., Kang L., Liu L. (2024). Trajectory optimization of vehicles at isolated intersection in a connected and automated environment. J. Transp. Eng. Inf..

[15] Zhao S. (2023). Research on Intelligent Control Method of Intersection Signal Light.

[16] Wan C., Hwang M. (2018). Value-based deep reinforcement learning for adaptive isolated intersection signal control. I Intell. Transp. Syst..

[17] Xu M., Li J., Zuo D. (2024). Signal Timing Optimization via Reinforcement Learning with Traffic Prediction. J. Syst. Simul..

[18] Zhang W., Yan C., Li X., Fang L., Wu Y.J., Li J. (2023). Distributed signal control of arterial corridors using multi-agent deep reinforcement learning. IEEE Trans. Intell. Transp. Syst..

[19] Zhu T., Li X., Fan W., Wang C., Liu H., Zhao R. (2021). Trajectory optimization of CAVs in freeway work zone considering car-following behaviors using online multiagent reinforcement learning. J. Adv. Transp..

[20] An M., Fan X., Cai H. (2020). Research on intelligent coordinated control of traffic light based on fog computing and reinforcement learning. Appl. Res. Comput..

[21] Zhou L., Leng S., Wang Q., Liu Q. (2023). Integrated Sensing and Communication in UAV Swarms for Cooperative Multiple Targets Tracking. IEEE Trans. Mob. Comput..

[22] Feng Y. (2022). Research on Trajectory Planning and V2X Communication Technology for Connected and Automated Vehicle Platoons.

[23] Lin X., Wang C., Wang K., Li M., Yu X. (2021). Trajectory planning for unmanned aerial vehicles in complicated urban environments: A control network approach. Transp. Res. Part C Emerg. Technol..

[24] Quan Q., Fu R., Li M., Wei D., Gao Y., Cai K.Y. (2022). Practical Distributed Control for VTOL UAVs to Pass a Virtual Tube. IEEE Trans. Intell. Veh..

[25] Tabares D. (2023). Aircraft ground airport operations automation. Proceedings of the 23rd AIAA Aviation Forum.

[26] Al-Msari H., Koting S., Ahmed A.N., El-Shafie A. (2024). Review of driving-behaviour simulation: VISSIM and artificial intelligence approach. Heliyon.

[27] Zhang K., Chang C., Wang S., Zhang Z., Li L. (2024). Review of autonomous vehicle simulators: Capabilities, challenges, and development directions. J. Transp. Eng. Inf..

[28] Lv L., Deng Z., Shao C., Shen W. (2023). A variable neighborhood search algorithm for airport ferry vehicle scheduling problem. Transp. Res. Part Emerg. Technol..

[29] Chen X., Li M., Lin X., Yin Y., He F. (2021). Rhythmic Control of Automated Traffic—Part I: Concept and Properties at Isolated Intersections. Transp. Sci..

[30] Chen X., Li M., He F. (2021). Multi-rhythm control for heterogeneous traffic and road networks in CAV environments. Transp. Res. Part Logist. Transp. Rev..

[31] Yuan D., Zhu X., Li Q., Tang Z., Qi M., Zou Y., Zhong Y., Zhao Y. (2024). Variational rhythm control for unmanned ground support vehicles on airport apron: Trajectory planning and roadside signal optimization. SSRN.

[32] Li Z., Zhu X., Zhang T., Chen H. (2023). Optimal Design of Push-back Spot in Single-aisle U-shaped Apron Area of Large Airports. Sci. Technol. Eng..

[33] Xu H., Qu J., Wang M., Zhu X. (2024). Research on optimization of task scheduling for forest fire rescue helicopter bucket firefighting. Fire Sci. Technol..

[34] Liu X., Mao W., Yang Q. (2024). A Resource Allocation Algorithm for Space-Air-Ground Integrated Network Based on Deep Reinforcement Learning. J. Electron. Inf. Technol..

[35] Yao X., Du Z., Sun Z., Calvert S.C., Ji A. (2024). Cooperative lane-changing in mixed traffic: A deep reinforcement learning approach. Transp. Transp. Sci..

